# What difference does FBT make? Results of a service audit following the implementation of FBT

**DOI:** 10.1186/2050-2974-2-S1-O17

**Published:** 2014-11-24

**Authors:** Liesje Donkin, Mysliwiec Roger, Maugan Rimmer

**Affiliations:** 1 Regional Eating Disorders Service, Auckland, New Zealand

## 

Manualised Family Based Treatment (FBT) is considered the best evidenced treatment for adolescents with Anorexia Nervosa. However, the replication of outcome data from research trials into treatment in a general clinical setting can be a challenge. Following on from the presentation entitled 'Overcoming challenges of implementation of FBT at the Regional Eating Disorders Service (REDS) in Auckland, New Zealand', the results of the implementation of the delivery of FBT at Auckland's Regional Eating Disorders Service (REDS) are presented. Results are based on a clinical audit of treatment outcomes in the year preceding the implementation of FBT. Data will be presented of the period of 18 months while FBT was established, and the 6 months afterwards. A comparison of these different periods will be made with respect to weight recovery and psychometrics as well as effects on length of admission and re-admission rate to the paediatric hospital.

This abstract was presented in the **Service Initiatives: Child and Adolescent Refeeding and FBT** stream of the 2014 ANZAED Conference.

